# The Role of Silicon in Antiherbivore Phytohormonal Signalling

**DOI:** 10.3389/fpls.2019.01132

**Published:** 2019-09-18

**Authors:** Casey R. Hall, Jamie M. Waterman, Rebecca K. Vandegeer, Susan E. Hartley, Scott N. Johnson

**Affiliations:** ^1^Hawkesbury Institute for the Environment, Western Sydney University, Penrith, NSW, Australia; ^2^York Environment and Sustainability Institute, Department of Biology, University of York, York, United Kingdom

**Keywords:** allelochemical, induced defences, insect, jasmonates, plant defence, silica, silicon

## Abstract

The role of plant silicon (Si) in the alleviation of abiotic and biotic stress is now widely recognised and researched. Amongst the biotic stresses, Si is known to increase resistance to herbivores through biomechanical and chemical mechanisms, although the latter are indirect and remain poorly characterised. Chemical defences are principally regulated by several antiherbivore phytohormones. The jasmonic acid (JA) signalling pathway is particularly important and has been linked to Si supplementation, albeit with some contradictory findings. In this Perspectives article, we summarise existing knowledge of how Si affects JA in the context of herbivory and present a conceptual model for the interactions between Si and JA signalling in wounded plants. Further, we use novel information from the model grass *Brachypodium distachyon* to underpin aspects of this model. We show that Si reduces JA concentrations in plants subjected to chemical induction (methyl jasmonate) and herbivory (*Helicoverpa armigera*) by 34% and 32%, respectively. Moreover, +Si plants had 13% more leaf macrohairs than −Si plants. From this study and previous work, our model proposes that Si acts as a physical stimulus in the plant, which causes a small, transient increase in JA. When +Si plants are subsequently attacked by herbivores, they potentially show a faster induction of JA due to this priming. +Si plants that have already invested in biomechanical defences (e.g. macrohairs), however, have less utility for JA-induced defences and show lower levels of JA induction overall.

## Silicon and Herbivore Defence

There has been increasing interest in the functional role of silicon (Si) in plant biology, with a strong recent focus on the mechanisms by which Si alleviates the effects of stress (Cooke et al., 2016; Debona et al., 2017; Coskun et al., 2019). Silicon is known to protect plants against a range of abiotic stresses, including drought, salt stress, toxic metals and nutrient deficiency (Ma, 2004; Liang et al., 2007; Guntzer et al., 2012), and biotic stresses, including pathogen infection (Van Bockhaven et al., 2013; Wang et al., 2017) and herbivory (Massey et al., 2007; Reynolds et al., 2009). Plants obtain Si *via* uptake of soluble silicic acid (Si(OH)_4_) from the soil and deposit it (as silica) within or between cells, in the cell wall or as discrete opaline phytoliths (Ma and Yamaji, 2006; Cooke and Leishman, 2011; Hartley et al., 2015). It is this deposition that likely underpins stress alleviation, although the exact mechanisms for stress alleviation remain controversial (Coskun et al., 2019).

In terms of herbivore defence, several mechanisms have emerged to explain how Si confers resistance to vertebrate and invertebrate herbivores (Massey and Hartley, 2006; Alhousari and Greger, 2018). Physical or biomechanical defences include discrete phytoliths or other abrasive structures (e.g. Si-fortified leaf trichomes), which can wear down insect mouthparts (Massey and Hartley, 2009) and may also prevent herbivores from processing ingested food efficiently resulting in inadequate nutrition (Massey and Hartley, 2006; Massey and Hartley, 2009). Silicon deposition within or between cell walls also makes cells more impact absorbent, harder to physically crush and less susceptible to fracture propagation (Clissold, 2008; Hunt et al., 2008).

Less well studied, Si has also been linked to the increased production of antiherbivore chemical defences in plant tissues (Alhousari and Greger, 2018). Silicon has a limited repertoire of chemical reactivity within plants, however; so it seems likely that this linkage is indirect and arises because of some other change associated with silicification (Coskun et al., 2019). We emphasise this point, and when we refer to Si having effects on defensive chemistry, we consider these to be indirect effects, and Si is not reacting with chemical pathways directly.

In particular, a number of studies involving plant pathogens point to Si stimulating production of defensive and oxidant enzymes, including peroxidase (POX), phenylalanine ammonia-lyase (PAL) and polyphenol oxidase (PPO) (Cherif et al., 1994; Liang et al., 2005; Fauteux et al., 2006; Cai et al., 2008; Rahman et al., 2015). These enzymes are also involved in induced resistance to herbivores, and several studies involving herbivores show similar effects of Si on defensive and oxidant enzyme activity. In particular, Si increased levels of PPO, POX and PAL in wheat plants inoculated with the aphid, *Schizaphis graminum* (Ranger et al., 2009). Similarly, Si increased the activities of catalase (CAT), PAL, POX, PPO and superoxide dismutase in rice (*Oryza sativa*) plants under attack by the leaf folder (*Cnaphalocrocis medinalis*) (Han et al., 2016). These defensive and oxidative enzymes are regulated by, and influence the regulation of, a number of defence-specific phytohormones including jasmonic acid (JA), salicylic acid (SA) and ethylene and ultimately the downstream defensive metabolites that affect herbivores (Howe and Jander, 2008). Once activated, chemical defences may be directly deterrent or harmful to the herbivore or involve recruitment of the herbivore’s natural enemies *via* volatile organic compound emission (Reynolds et al., 2016). In general, JA regulates defences against tissue-chewing insects (Erb et al., 2012), whereas defences against phloem-feeding insects are regulated by both SA and JA (Züst and Agrawal, 2016).

## Silicon and Antiherbivore Phytohormonal Signalling

To date, our understanding of how Si affects antiherbivore phytohormonal signalling derives entirely from two studies in rice. These studies, published roughly at the same time, led the way in demonstrating that Si had significant impacts on the JA pathway in particular (Ye et al., 2013; Kim et al., 2014). Using authentic herbivory and chemical induction with methyl jasmonate (MeJA), Ye et al. (2013) demonstrated that Si stimulated an increase in the activity of several defensive enzymes, including PPO and POX. Using mechanical wounding, Kim et al. (2014) demonstrated similar effects of Si on POX, PPO and CAT. Strikingly, however, these studies showed opposing effects of Si on the JA pathway in damaged plants; Ye et al. (2013) reported increased JA levels, whereas Kim et al. (2014) reported JA levels were reduced by Si. These findings were interpreted differently. Ye et al. (2013) concluded that there was a stimulatory effect of Si on JA activity following damage, which increased levels of defensive enzymes such as those that scavenge the potentially harmful reactive oxygen species (ROS) arising from tissue damage. In contrast, Kim et al. (2014) suggested that the higher levels of defensive enzymes in Si-treated plants caused a reduction of ROS arising from wounding and therefore suppression of an early signalling event for JA production. Another difference was that in damage-free plants Si addition caused a small but significant increase in JA levels (Kim et al., 2014), subsequently confirmed by Jang et al. (2018), whereas Ye et al. (2013) saw no such increase.

The study by Ye et al. (2013) has proven influential, and subsequent reviews have generally adopted the narrative that Si promotes the activity of the JA pathway in plants under herbivore attack (e.g. Reynolds et al., 2016; Debona et al., 2017; Alhousari and Greger, 2018). There is also at least one subsequent empirical study that indicates a positive linkage between Si and JA (Liu et al., 2017). The mechanism by which Si affects JA-induced herbivore defences is unknown but, as already mentioned, silica (the polymerised form) has limited capacity for biochemical activity, so it is hard to envisage how it would directly modify the chemistry of phytohormonal signalling pathways. Frew et al. (2018) and Coskun et al. (2019) suggested that defences in Si-treated plants may be higher because Si in the apoplast may physically interfere with effector molecules released by the herbivores that would otherwise suppress plant defence responses (Hogenhout and Bos, 2011). In other words, the defence response in the +Si plants is no longer compromised by the herbivore.

## Observations From *Brachypodium Distachyon*

Our objective in this Perspectives article is to stimulate further research into the relationship between Si and antiherbivore phytohormonal signalling. Given that this has, to our knowledge, only been studied in rice, it may be timely to address whether the same mechanisms operate in noncrop grasses. In particular, we report findings from two experiments conducted under different conditions that tested whether Si supplementation of the model grass *Brachypodium distachyon* changed JA levels in response to chemical (MeJA) and herbivore (*Helicoverpa armigera*) treatments (Experiments 1 and 2, respectively). Full details of the materials, experimental procedures and chemical analyses are given in **Supplementary Material**. In brief, plants were either supplemented with potassium silicate (+Si) or nonsupplemented (−Si). Half of the +Si and −Si plants were then subjected to chemical (Experiment 1) and herbivore (Experiment 2) treatments. We then quantified foliar concentrations of JA and Si in all plants. Leaf macrohair density was additionally quantified in Experiment 2. Two-way analyses of variance were conducted in all cases.

**Figure 1:**
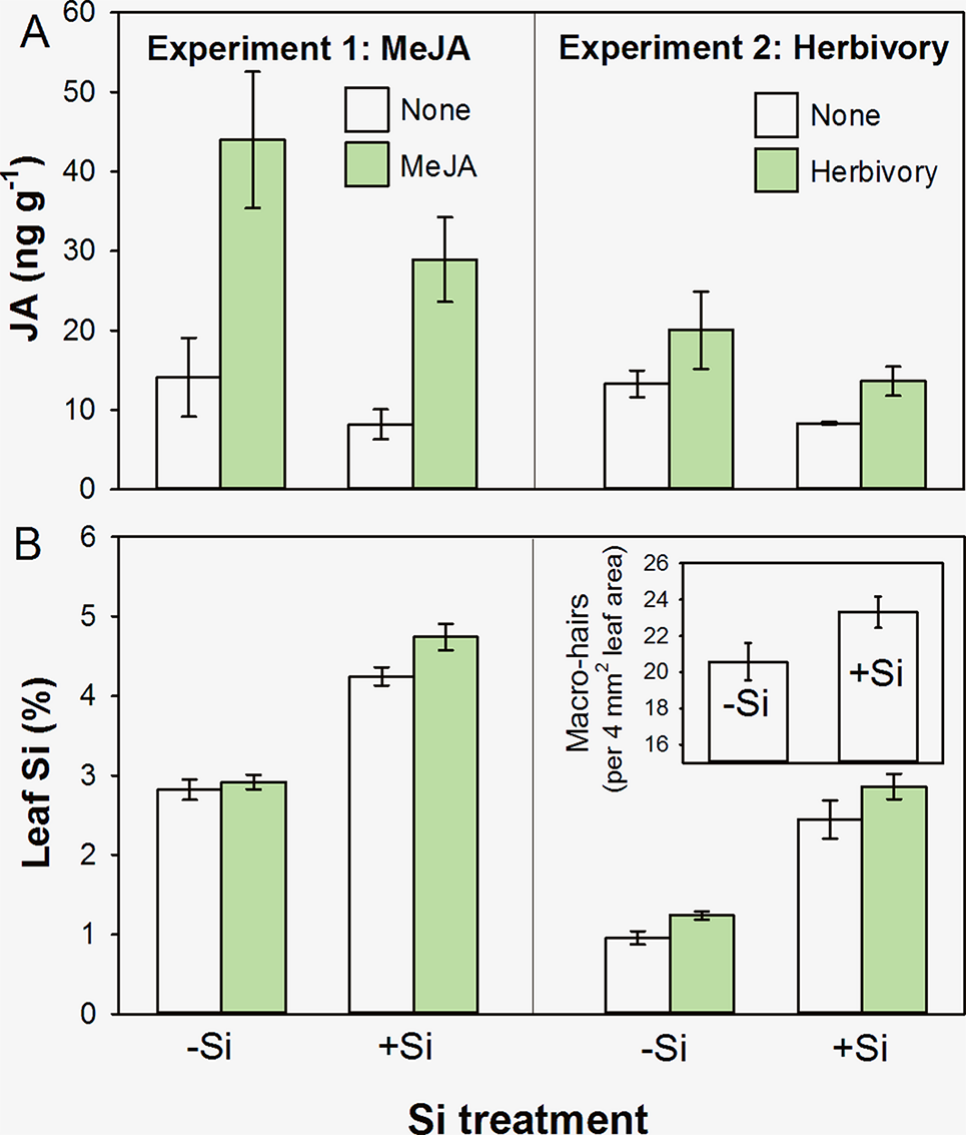
Foliar concentrations of **(A)** JA and **(B)** Si in *B. distachyon* plants that had been either supplemented (+Si) or nonsupplemented (−Si) with Si and subsequently subjected to exogenous application of MeJA (Experiment 1) or herbivory by *Helicoverpa armigera* (Experiment 2). Figure inset in Experiment 2 shows leaf macrohair density on −Si and +Si plants. Mean ± standard error shown in all cases.

We found that concentrations of JA were reduced in +Si plants in both experiments (**Figure 1A**), although this was marginal (i.e. not significant at a 95% confidence interval) in Experiment 1 (*F*_1,19_ = 3.82, *P* = 0.066) but was in Experiment 2 (*F*_1,20_ = 7.90, *P* = 0.011). Methyl jasmonate and herbivory caused significant increases in JA (**Figure 1A**; *F*_1,19_ = 31.05, *P* < 0.001, and *F*_1,20_ = 6.60, *P* = 0.018, respectively). There was no statistically significant interaction between Si treatment and the ‘inducing agent’ in either experiment reflecting that JA was being induced in both +Si and −Si plants (*F*_1,19_ = 0.47, *P* = 0.501, and *F*_1,20_ = 0.53, *P* = 0.475, respectively).We found a consistent pattern in JA levels in both cases; however, Experiment 2 tended to have lower overall levels of JA. This could be due to the timing of sampling; plant material was sampled after a week of herbivory as opposed to 24 h after MeJA treatment in Experiment 1. In addition, plants in the second experiment were grown under short photoperiod to avoid flowering, and disruption of circadian rhythms can lead to lower JA levels (Cagnola et al., 2018). The consistency of the JA response across several different experiments with differing conditions, however, suggests that Si plays a role in the JA response.

As might be anticipated, Si supplementation resulted in higher foliar concentrations of Si in both experiments (**Figure 1B**; *F*_1,44_ = 161.35, *P* < 0.001, and *F*_1,27_ = 117.85, *P* < 0.001, respectively). Application of MeJA (Experiment 1) and herbivores (Experiment 2) resulted in increased levels of Si, indicative of an induced Si defensive response (**Figure 1B**; *F*_1,44_ = 5.37, *P* = 0.025, and *F*_1,27_ = 5.54, *P* = 0.026, respectively). Again, there was no statistically significant interaction between Si treatment and the ‘inducing agent’ in either case (*F*_1,44_ = 2.44, *P* = 0.125, and *F*_1,27_ = 0.20, *P* = 0.660, respectively).

In Experiment 2, we also observed that Si promoted the formation of nonglandular macrohairs (see **Figure 1B** inset; *F*_1,25_ = 4.56, *P* = 0.043). This is compatible with the findings of Glazowska et al. (2018), who showed that a low-silicon accumulating *B. distachyon* mutant had fewer and shorter leaf macrohairs. We consider this to be the likely explanation for why we saw decreases in JA in +Si plants that were physically better defended with nonglandular leaf macrohairs (or trichomes). In hydroponics systems, we observed that relative growth rates of *H. armigera* declined by more than 150%, and relative consumption decreased by 58% when feeding on +Si plants compared to −Si plants (*t*_9_ = 3.2, *P* = 0.008, and *t*_9_ = 2.3, *P* = 0.04, respectively) (Hall et al., submitted). Nonglandular trichomes are widely reported to have antiherbivore properties (Werker, 2000), and their production has been linked to Si uptake (McLarnon et al., 2017). Whereas the formation of glandular trichomes is regulated by JA, nonglandular trichomes or macrohairs can be formed independently of the JA pathway. In particular, a *COI*-deficient (JA-insensitive) rice mutant had significantly fewer glandular trichomes but nonglandular trichomes developed normally (Li et al., 2004).

**Figure 2:**
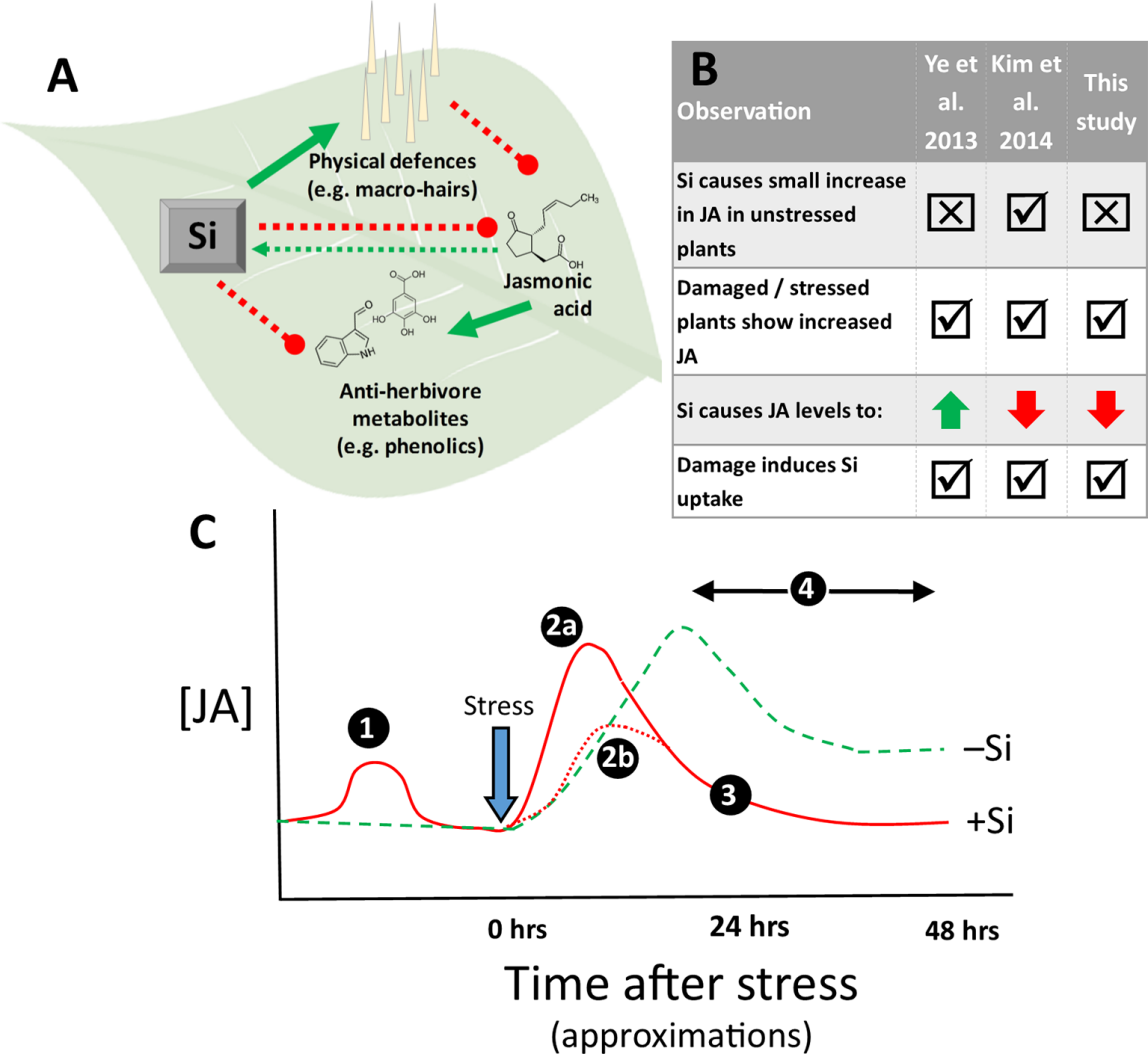
Schematics showing **(A)** the hypothesis for *B. distachyon*, **(B)** key similarities and differences between studies and **(C)** a proposed conceptual model. In **(A)**, we hypothesise that Si directly promotes physical defences (solid green arrow), which indirectly dampens JA activity (dashed red lines). Herbivory still triggers the JA pathway, which could lead to synthesis of antiherbivore metabolites (solid green arrow), but in Si-enriched *B. distachyon*, this leads instead to further Si uptake (dashed green arrow), which indirectly suppresses metabolite production (dashed red line). In **(C)**, we propose a conceptual model for JA activity in Si accumulating (+Si, red lines) relative to nonaccumulating (−Si, green lines) plants. Example time points are given as indicative approximations. If Si supply acts as a mild form of stress that results in minor induction of the JA pathway in +Si plants (Stage 1) (e.g. Kim et al., 2014; Jang et al., 2018), this may result in two different scenarios (Stage 2a): subsequent stress (e.g. herbivory) causes a faster and/or greater JA response in these plants as a result of this priming (e.g. Ye et al., 2013). Alternatively, plants already invested in physical defences (e.g. our observations) or having higher existing levels of oxidative stress enzymes have less utility for JA-induced defences and therefore show muted JA induction (Stage 2b) and have lower levels of JA than −Si plants overall (Stage 3). The observation of Ye et al. (2013) that JA began to decline in +Si plants after an initial spike could align also with Stage 3, although this remains speculative.

We hypothesise that Si-supplemented *B. distachyon* deploy constitutive physical defence in the form of nonglandular macrohairs as an alternative to costly JA-induced chemical defences. There is extensive evidence that Si defences are negatively associated with phenolic defences (Cooke and Leishman, 2012; Frew et al., 2016; Johnson and Hartley, 2018), potentially due to a defensive trade-off, so this hypothesis is compatible with these observations. As summarised in **Figure 2A**, we hypothesise that Si promotes physical defences which dampens the JA response and production of antiherbivore metabolites, but herbivore induction of the JA pathway (albeit at lower levels) leads to further Si uptake. Silicon deposition in tissues is irreversible, so once in place, physical defences are independent of the JA pathway, and plants do not require further stimulation of this pathway (as might occur with sustained production of antiherbivore metabolites; **Figure 2A**).

## Similarities and Differences Between Observations

Our observations have some similarities and some differences with the studies by Ye et al. (2013) and Kim et al. (2014), as shown in **Figure 2B**. Some of these differences could simply arise because of differences in experimental approaches (e.g. hydroponic vs. soil experiments and timing of treatments), but there were some consistencies between studies. In all cases, damage increased levels of JA and induced Si uptake. In common with our observations (**Figure 1A**), Ye et al. (2013) did not detect a significant increases in JA in nonstressed plants, whereas Kim et al. (2014) reported a significant increase in JA (as did Jang et al., 2018). It is conceivable that Si may be perceived as mild stress due to silica bodies being deposited in the intercellular space and cell wall, which could cause a slight stimulation of antiherbivore defences (Ye et al., 2013). The presence of Si has been associated with the activation of oxidative enzymes known to stabilise ROS levels within plant tissues (Ye et al., 2013; Kim et al., 2014; Howladar et al., 2018). Although an overaccumulation of ROS may result in cellular damage, ROS are an integral component to a multitude of metabolic signalling processes (e.g. activation of the JA pathway) and are essential for growth in aerobic organisms (Mittler et al., 2004; Bailey-Serres and Mittler, 2006; Foyer and Noctor, 2013; Mittler, 2017). Furthermore, Kim et al. (2014) report slight increases in lipid peroxidation in ‘unstressed’ plants treated with Si, supporting the notion the Si may modify cellular redox biology. Nevertheless, the effects of Si on oxidative mechanisms vary depending on the type of stress, and Si does not ubiquitously increase activity of oxidant enzymes (Kim et al., 2017).

Timing could be important in this regard because it may be that this induction of JA dissipates as the presence of Si does not further stress the plant beyond baseline levels, in contrast to herbivory, which would sustain damage and therefore stress. It therefore may be that measurements of JA were taken too soon after damage treatments (e.g. 24 h; Experiment 1 and Ye et al., 2013) or too late after initial damage (e.g. 7 days; Experiment 2) to detect JA increases in unstressed +Si plants.

Kim et al. (2014) took measurements only 30 min after damage treatments and were able to detect differences between Si treatments in unstressed plants, suggesting that the differences might be short lived and occur early on in the defence response. Additionally, Kim et al. (2014) supplemented the plants with Si only 24 h before the experiment, whereas plants were exposed to Si for a minimum of 2 weeks in Experiment 1, Experiment 2, and Ye et al., 2013 (2, 12 and 3 weeks, respectively). It is not possible, therefore, to discount the possibility that the timing of Si exposure plays a role in the variation in responses between studies. The major difference between the results of these studies is that our observations, together with Kim et al. (2014), found that JA was lower in damaged plants treated with Si, regardless of whether the ‘damage’ was applied mechanically, chemically, or through authentic herbivory. In contrast, Ye et al. (2013) found a faster induction of JA in +Si plants 9 h after MeJA and herbivore treatment, which then declined. Studies that ‘standardise’ herbivore damage using simulated herbivory and associated cues (e.g. oral secretions) could help resolve such differences (Waterman et al., 2019).

## A Conceptual Model For Si-Ja Relationships

Based on findings to date, we propose a model for how Si may relate to antiherbivore phytohormonal signalling, focusing on the JA pathway. It cannot be comprehensive given the limited information currently available but is intended to act as a framework for further hypothesis testing. We propose that Si can act as a physical stimulus in the plant, which triggers a small and transient increase in JA signalling in +Si plants (Stage 1 in **Figure 2C**). Critical plant signalling molecules (e.g. ROS) may be kept at marginally higher levels in unstressed +Si; thus, when +Si plants are subjected to a stress (i.e. herbivory), they might be primed for a faster induction of the JA pathway, so that a defensive response can be mounted more rapidly (Stage 2a in **Figure 2B**). −Si plants show a similar induction in JA levels but at a slower rate. Consistent with this, we note that stressed −Si plants in the study by Ye et al. (2013) had the highest levels of JA at 24 h (albeit not significantly different from +Si plants), which may have increased further over a longer time period. We propose that plants with adequate defences against herbivory in place (e.g. physical defences in *B. distachyon*) do not show this level of JA induction (Stage 2b in **Figure 2C**) and as a consequence will have lower levels of JA overall (Stage 3 in **Figure 2C**). This is also consistent with the argument that Kim et al. (2014) advance that +Si plants have greater mechanical strength (the lipid membrane is more rigid), and therefore, stress from wounding is less pronounced.

## Conclusions And Unresolved Issues

Although it has been shown that Si may alter redox chemistry (e.g. through regulating enzymatic oxidants), the precise mode of action remains ambiguous. Ye et al. (2013) proposed that silica bodies deposited in the intercellular space and cell wall may be perceived by the plant as a minor stress, potentially resulting in the accumulation of plant defence signals (e.g. ROS and JA). This hypothesis is further supported by findings that Si, without the presence of an additional stressor, showed higher lipid peroxidation, which likely suggests a higher level of ROS under baseline (unstressed) conditions (Kim et al., 2014). However, due to inconsistencies in defence signalling between studies, for example, up- and down-regulation of JA under no stress conditions, the role of Si in mediating defence signals remains elusive. It is possible that these discrepancies are in part due to inconsistencies in the timing of not only measurements but also exposure to Si. Additional experiments that explore potential temporal variation in Si-induced defence responses are required.

Certain grass species may have evolved the capacity to hyperaccumulate Si as a defensive response to JA signalling in lieu of extensive arrays of JA-induced chemical defences, which are metabolically costly. This would be consistent with our model that proposes that Si-enriched plants are possibly capable of faster JA responses, but show lower levels of JA induction overall as they have extant biomechanical or physical protection in place (**Figure 2A**). In support of this, a broad range of plants with Si defences produce fewer phenolic and tannin defences (Cooke and Leishman, 2012). Leaving Si aside, many plants have evolved the capacity to hyperaccumulate metalloids for antiherbivore defence while producing fewer antiherbivore metabolites (see Defensive Enhancement Hypothesis; Boyd, 2012). It therefore seems plausible that Si could play an analogous role in antiherbivore phytohormonal signalling.

## Data Availability Statement

The datasets generated for this study are available on request to the corresponding author.

## Author Contributions

The experiments were conceived by CH, RV, SH and SJ and executed by CH and RV. CH and SJ analysed and interpreted the data. The wider paper was conceived by SJ with all authors (SJ, CH, RV, JW and SH) making significant contributions.

## Funding

This research was funded by an Australian Research Council Future Fellowship awarded to SJ (FT17 0100342) and Discovery project awarded to SJ and SH (DP170102278).

## Conflict of Interest

The authors declare that the research was conducted in the absence of any commercial or financial relationships that could be construed as a potential conflict of interest.
