# Supplementary material for: The Role of Silicon in Antiherbivore Phytohormonal Signalling
Source: Front Plant Sci. 2019 Sep 18;10:1132. doi: 10.3389/fpls.2019.01132 (PMC6759751; doi:10.3389/fpls.2019.01132)
Supplement: Supplementary file 1 [file Table_1.docx]

**Supplementary Material**

*Plant material and Si treatments*

*Brachypodium distachyon* (Bd21-3) plants were grown from seed (supplied by INRA, Versailles, France) in soil recovered from the Hawkesbury Campus of Western Sydney University (33.6138 ºS, 150.7500 ºE). Seed was initially stratified for 5 days at 4ºC before planting. Plants were grown in a 50:50 composite of sandy loam and loam soil with low bioavailable Si (16.00 ± 3.46 mg kg^-1^). In both experiments, half of the plants (selected at random) were assigned for Si supplementation (+Si plants) and the remaining plants assigned for non-supplementation (–Si plants). +Si plants were watered with a solution consisting of potassium silicate (Agsil32, PQ Australia, SA, Australia) at a concentration of 2 mM (SiO_2_ equivalent) and adjusted to pH 7 using HCl. –Si plants were watered with a control solution containing 1.6 mM KCl to balance additional K+ and Cl- in the +Si treatments.

Experiment 1 – *MeJA Study*

The experiment comprised a factorial combination of Si supplementation (+/–) and application of MeJA (+/–) replicated 12 times. Plants were grown in a 3 m x 5 m x 3 m glasshouse chamber with UV transparent plexiglass (6 mm thick) walls and roof and were naturally lit throughout the experiment. Air temperature was maintained at 22/18°C day/night on a 14 L:10 D cycle. Humidity was controlled at 50% (±6%). Plants were rotated weekly within the chamber. Silicon treatments started two weeks after germination where each plant received 50 mL of either +Si or -Si solution every three days for two weeks. At four weeks after germination plants were sprayed with 1 mM MeJA solution prepared with 0.01% Tween 20, or 0.01% Tween 20 in water (control plants). Plants were sampled for JA quantification 24 hours after MeJA application. Plants were frozen in liquid nitrogen and stored at -80°C until freeze dried. Three days after MeJA application all remaining plants were harvested and dried at 40°C for Si analysis. We have found that three days post MeJA treatment was enough time for Si to accumulate in plant tissues in response to the initial spike in JA.

*Experiment 2 – Herbivory Study*

The experiment comprised a factorial combination of Si supplementation (+/–) and application of herbivores (+/–) replicated 10 times. Plants were grown in a growth cabinet (Climatron, Thermoline Scientific) maintained at 22ºC on a 8:16 hour light:dark cycle to prevent flowering. Humidity was kept at c. 60% and light levels were ca. 150 μmol m^-2^ s^-1^. Si treatment started two weeks after germination. Plants were watered with 25mL of either +Si or –Si solutions (20 plants in each case). Solutions were applied every 10 days for the following eight weeks and then every 7 days for the remaining four weeks of the experiment for a total of 12 weeks of Si treatment. Plants were grown for a total of 14 weeks before being subjected to herbivory. Approximately 100 *Helicoverpa armigera* eggs (supplied by CSIRO Agriculture & Food, Narrabri, Australia) were individually hatched on growing media (modified from Teakle and Jensen, 1985). Upon reaching fourth instar (c. 14 days later), one herbivore was applied to each plant, using half of the –Si and +Si plants. To retain herbivores on individual plants, transparent plastic cages (175 🞨 100 🞨 50 mm; 650 mL) with ventilation were applied to all plants (including herbivore-free plants). Herbivores were removed after one week and plant tissue immediately frozen in liquid nitrogen and stored at -80°C until freeze dried. Prior to harvesting, three leaves without herbivore damage were selected from each plant at random, magnified 40🞨 and photographed. Trichomes (all were non-glandular) on the adaxial leaf surface were quantified from a 2🞨2 mm (4 mm^2^) section of leaf selected at random.

*Analysis of Si and JA*

Approximately 80mg of ground leaf material was analysed to measure Si concentration using an X-ray fluorescence spectrometer (Epsilon 3*^x^*; PANalytical, EA Almelo, The Netherlands) following the procedure of Reidinger et al. (2012). Analysis was calibrated using citrus plant material (NCS ZC73018 Citrus leaves, China National Institute for Iron and Steel) of known Si concentrations (see Hiltpold et al., 2017 for full details). Deuterated jasmonic acid (D5-JA) internal standard was purchased from CDN Isotopes (Quebec, Canada). HPLC grade methanol, chloroform, and phytohormone calibration standards were purchased from Sigma-Aldrich (MO, USA).

All harvested leaves were immediately frozen in liquid nitrogen and stored at -80°C until freeze dried for further analysis. Jasmonic acid (JA) was extracted using the Bligh-Dyer method (Bligh and Dyer, 1959) to remove interfering compounds from the plant matrix. In brief, 50 mg of ground leaf material was extracted with 500 uL of 70% methanol spiked with D5-JA as internal standard to yield a final concentration of 100 ppb. Samples were mixed for 30min at 4ºC in a rotator mixer, 180 uL of chloroform was added and samples vortexed for 30 s. This was repeated with another 180 uL of chloroform and then 200 uL of water was added, samples were then centrifuged at 6000 rpm for 10 min at room temperature, resulting in a triphasic system. The upper water/methanol layer was carefully transferred to a clean 2 ml Eppendorf tube and passed through a 0.22 µm PTFE filter. The extracts were analysed by UPLC/ESI-MS/MS using an Acquity UHPLC coupled to a Xevo triple quadrupole mass spectrometer (Waters Corporation, Milford, USA). Five microliters of extract were injected into a 2.1 mm x 50 mm x 1.7 µm, C18 reverse phase column. The mobile phase was composed of water (A) and acetonitrile (B) both containing 0.1% (v/v) formic acid at a constant flow rate of 0.6 ml min^-1^. Elution was performed as a linear gradient (A%, *t* min): 80% A at 0 min; 50% A at 2 min; 0% A and 2.1 min. Jasmonic acid was detected by ESI-MS/MS operating in negative ion mode. JA identification was based on the fragmentation pattern as compared with an authentic standard. Quantification was based on a calibration curve of the standard and adjusted for sample recovery based on the internal standard. Final concentrations were standardised by dry weight of the sample.

*Statistical analysis*

Plant chemistry was analysed with an unbalanced ANOVA due to some replicates being excluded where herbivores had prematurely escaped or low sample recovery leading to unreliable JA results. The two-way ANOVA included Si treatment and the ‘inducing agent’ presence (i.e. MeJA or herbivory) as individual and interacting factors. Trichome counts were of herbivore-free plants were analysed with a one-way ANOVA (Si treatment) with plant number included as a random factor to account for measurements being taken from three leaves per plant. To meet assumptions of normality, JA data had Johnson transformations applied prior to analysis (Johnson, 1949; Chou et al., 1998). Analysis was conducted in Genstat (version 18; VSN International, Hemel Hempstead, UK).

**References**

Bligh, E.G., and Dyer, W.J. (1959). A rapid method of total lipid extraction and purification. *Can. J. Biochem. Physiol.* 37, 911-917. doi:

Chou, Y.M., Polansky, A.M., and Mason, R.L. (1998). Transforming non-normal data to normality in statistical process control. *J. Qual. Technol.* 30, 133–141. doi: 10.1080/00224065.1998.11979832

Hiltpold, I., Demarta, L., Johnson, S.N., Moore, B.D., Power, S.A., and Mitchell, C. (2017). "Silicon and other essential element composition in roots using X-ray fluorescence spectroscopy: a high throughput approach", in: *Invertebrate Ecology of Australasian Grasslands. Proceedings of the Ninth ACGIE.* (ed.) S.N. Johnson. (Hawkesbury, NSW, Australia: Western Sydney University).

Johnson, N.L. (1949). Systems of frequency curves generated by methods of translation. *Biometrika* 36, 149-176. doi: 10.2307/2332539

Reidinger, S., Ramsey, M.H., and Hartley, S.E. (2012). Rapid and accurate analyses of silicon and phosphorus in plants using a portable X-ray fluorescence spectrometer. *New Phytol.* 195, 699-706. doi: 10.1111/j.1469-8137.2012.04179.x

Teakle, R.E., and Jensen, J.M. (1985). "*Heliothis punctiger*," in *Handbook of insect rearing, vol. 2,* eds. R. Singh & R.F. Moore. (Amsterdam, The Netherlands: Elsevier), 312-322.
